# Tibial lengthening in congenital pseudoarthrosis of the tibia: a scoping review

**DOI:** 10.1186/s12891-026-09666-4

**Published:** 2026-03-06

**Authors:** Siddarth K. Kamath, Arkesh Madegowda, Anuj Baghel, Kumar Amerendra Singh, Hitesh H. Shah

**Affiliations:** 1 Department of Paediatric Orthopaedics Kasturba Medical College, Manipal Academy of Higher Education (MAHE), Manipal, Karnataka India; 2 Department of Orthopaedics, All India Institute of Medical Sciences, Raipur, India

**Keywords:** Congenital pseudoarthrosis tibia, Tibial lengthening, External fixator, Distraction osteogenesis

## Abstract

**Background:**

Congenital pseudoarthrosis of the tibia (CPT) is a rare, sinister condition that is often associated with neurofibromatosis type 1 (NF1). Even after achieving union, patients frequently present with significant leg length discrepancy (LLD) due to growth disturbance and repeated reconstructive surgeries. Tibial lengthening using distraction osteogenesis is commonly performed, yet outcomes and complications remain poorly understood.

**Objective:**

To systematically map the available literature on tibial lengthening in CPT, describe surgical techniques, summarize reported outcomes, and identify research gaps.

**Methods:**

A scoping review was conducted following Arksey and O’Malley’s framework and the PRISMA-ScR guidelines. The PubMed, Embase, Scopus, Web of Science and Cochrane Library databases were searched. Eligible studies included those reporting tibial lengthening during CPT. Data on patient demographics, surgical techniques, length achieved, union rates, complications, and follow-up were extracted.

**Results:**

Thirty-one studies involving 486 patients were included. All studies were retrospective. The mean age at lengthening ranged from 3.2 to 33.7 years. NF1 was present in 70% of the patients. The Ilizarov external fixator in isolation or with hybrid techniques was the most frequently reported lengthening modality. The mean lengthening achieved was 5.5 cm (range 1.5–10.2 cm). The most common site of lengthening was through proximal tibial osteotomy followed by physeal distraction. Complications such as pin tract infection, regenerate fractures and tibial bowing deformities were commonly encountered. The follow-up period ranged from 1.6 to 24 years.

**Conclusions:**

Tibial lengthening is feasible in CPT and can achieve meaningful limb length equalization. However, complication rates remain high, and evidence is limited by the small number of retrospective studies. Standardized outcome reporting and prospective multicenter collaboration are needed to optimize care.

**Supplementary Information:**

The online version contains supplementary material available at 10.1186/s12891-026-09666-4.

## Introduction

Congenital pseudoarthrosis of the tibia (CPT) is a rare, chronic, debilitating condition in paediatric orthopaedics with an incidence ranging from 1 in 1,40,000–2,50,000 live births. This condition is commonly associated with neurofibromatosis type 1 (70–80%). CPT is characterized by the presence of dysplastic bone and periosteum in the tibia, leading to problems such as anterolateral bowing, pathological fracture and nonunion at the fracture site [1, 2]. Various methods have been described in the literature to achieve union at nonunion sites, such as excision of the hamartoma with intramedullary rodding and cortical grafting, vascularized fibula grafting and external fixation, with variable rates of successful union [1, 3–5].

Despite advances in these methods, tibial shortening eventually results from the excision of bone fragments during surgery or from aberrant physeal growth [6]. This shortening adversely affects the child, causing problems in gait, pelvic obliquity, scoliosis, normal-side hip subluxation and impairment in activities of daily living [6]. The management of tibial shortening with lengthening of the involved tibia by distraction osteogenesis using external fixators (uniplanar or ring fixator) and intramedullary nails has become a vital component of the overall management of this complex condition [1, 7]. However, these methods are technically demanding and are fraught with complications such as pin tract infections, refractures and delayed healing of regenerate [1, 6, 8].

In the literature reviewed, several authors have described the general management of CPT. There are significant gaps in the literature regarding the age at which lengthening was performed, techniques used for lengthening, healing rates, sites of lengthening in the tibia and rates of distraction. Hence, this scoping review was undertaken to examine current evidence to address these questions.

A preliminary search of MEDLINE, the Cochrane Database of Systematic Reviews and JBI Evidence Synthesis was conducted, and no current or underway systematic reviews or scoping reviews on the topic were identified.

### Objectives of the study

To map and systematically synthesize evidence on tibial lengthening in CPT, including techniques, outcomes and complications.

## Methods

This scoping review was developed following Arksey and O’Malley’s framework and adhered to the Preferred Reporting Items for Systematic Reviews and Meta- Analyses extension for Scoping Reviews (PRISMA-ScR) guidelines [9, 10].

A peer-reviewed protocol was prepared a priori and has been registered on open science framework (registration link: 10.17605/OSF.IO/Y86DU). The methodology encompassed the following steps: (1) formulating the research question; (2) identifying pertinent studies; (3) selecting the studies; (4) charting the data; and (5) collating, summarizing, and reporting the findings.

### Formulating the research question

The research questions for this scoping review were as follows:

1. What was the age at which tibial lengthening surgery was performed?
2. What was the quality of regenerate formed and time to regenerate healing?
3. What was the amount and number of lengthening surgeries performed?
4. What were the techniques used to perform the lengthening?
5. What are the sites in tibia where lengthening was performed?

### Identifying pertinent studies

A comprehensive literature search was performed using electronic databases, including PubMed, Scopus, Web of Science, Embase and Cochrane Central until June 2025, and an additional search was performed in October 2025 to identify new studies.

The concepts used to develop the search string were “tibial lengthening” AND “congenital pseudoarthrosis”.

The search string used for PubMed was (“congenital pseudoarthrosis tibia” OR “congenital pseudoarthrosis” OR “congenital pseu*arthrosis”) AND (“tibial lengthening” OR “limb lengthening” OR “tibial length equalization” OR “Ilizarov technique” OR “distraction osteogenesis”). This search string was modified accordingly for other databases (Supplementary material 1).

Studies published from database inception through October 2025 were considered.

#### Inclusion criteria

- Studies including children and adults with unilateral congenital pseudoarthrosis of the tibia.
- Studies mentioning tibial lengthening surgery performed using an external fixator (monolateral or ring fixator) or intramedullary nail-assisted lengthening techniques.
- Studies including osteotomy and bone transport of the tibia.
- Studies mentioning vascularized bone grafts with lengthening were also be included.

#### Exclusion criteria

- Case reports and case series with fewer than five cases were excluded to improve data consistency.
- Publications not containing primary data such as review articles, editorials and book chapters.
- Conference abstracts due to incomplete data and redundancy.
- Articles not written in English language.

No additional filters were applied during the search to ensure inclusivity.

### Selecting the studies

**Fig. 1:**
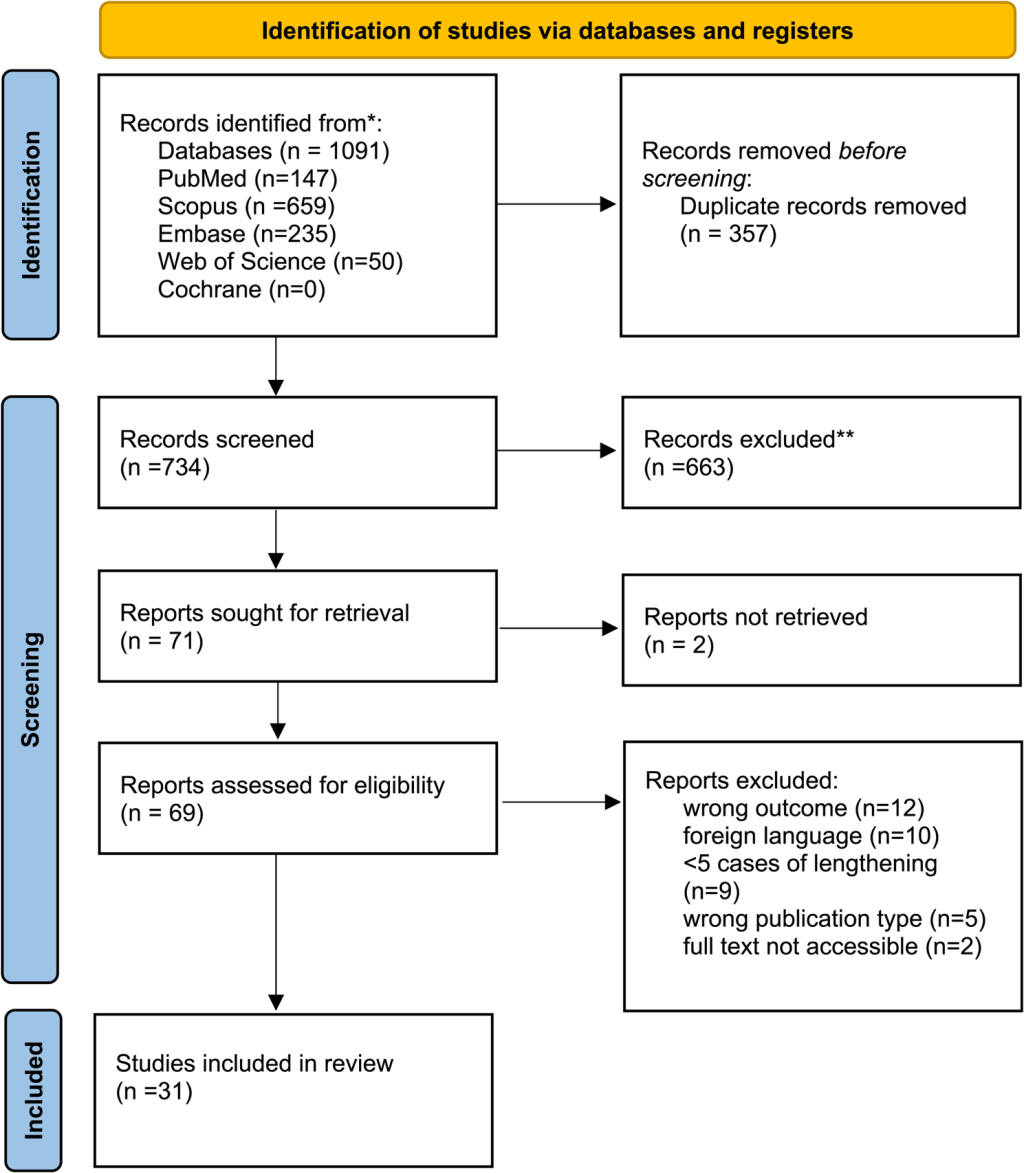
(PRISMA flowchart)

All identified citations were collected and uploaded into Rayyan (Qatar Computing Research Institute, Doha, Qatar), where duplicates were removed. The deduplication tool in Rayyan was set at 95% similarity between articles to consider duplicates. The selection process began with the removal of duplicate records from different databases. Titles and abstracts were then screened to evaluate their eligibility, followed by a thorough review of complete texts by three researchers (SKK, AM, AB). Potentially relevant sources were retrieved in full, and their citation details were imported. The references of the articles included in the full-text screening were examined to prevent omissions. Disagreements regarding inclusion or exclusion were resolved through consensus, with the assistance of another researcher (HHS). The article selection process has been summarized in the PRISMA flow chart (Fig. 1).

### Charting the data

Three authors (SKK, AM, AK) used a standard Excel data charting format to extract relevant information from the selected studies (supplementary material 2-data proforma).

The following details were extracted from the articles that were included in the study:

• Study details: author, year, study period and study design.
• Patient demographics: Number, age at which lengthening was performed and NF1 association.
• Intervention: type of lengthening device/technique, osteotomy site and rate of distraction.
• Outcomes:
  ◦ Length achieved.
  ◦ Healing index: time taken for consolidation per centimeter of regenerate/days.
  ◦ Latency period: Time interval between the tibial osteotomy and beginning of lengthening.

Complications.

### Collating, summarizing, and reporting the findings

Owing to the heterogeneity of the studies involved and the variability in reported outcomes, the scoping review approach was adopted instead of a systematic review, as it allowed for a broad examination of available evidence without imposing strict methodological constraints. Data extracted from the articles were summarized and reported. A quality appraisal was not performed on the studies included, as the objective of this scoping review was to map the literature rather than select the best evidence.

## Results

### Study selection

A total of 1091 records were identified from the database searches. After the removal of duplicates (*n* = 357), 734 records were screened by title and abstract. Of these, 71 full texts were reviewed, and 31 studies met the inclusion criteria. A PRISMA flow diagram (Fig. 1) illustrates this process.

Tables 1, 2, 3, 4, 5 and 6 summarize studies investigating the types of interventions used and the outcomes of tibial lengthening in CPT.

**Table 1:** Summary of extracted data

Author	Agashe et al [1]	Balci Hİ et al [6]	Cho TJ et al [3]	Eamsobhana P et al [8]	El-Gammal TA et al [4]
Year	2012	2021	2007	2010	2023
Region	Korea	Turkey	Korea	Thailand	Egypt
Design	Retrospective	Retrospective	Retrospective	Retrospective	Retrospective
Duration	2003–2008	1997–2016	NM	1996 to 2005	1996 to 2017
N	15 (BT/L-NM)	27 (BT None; L 27)	22 (27 lengthenings) (BT None; L 27)	13 (BT/L not mentioned)	11 (BT None; L 11)
Age of lengthening	7.5 years	5.74 years	7 years	7 years	10.2 years
Subgroups	NA	NA	NA	NA	NA
NF-1	7	18	NM	5	11
Preop shortening (Mean)	2.5 cm	5.81 cm	NM	NM	7.9 cm
Fixation method	Ilizarov + Intramedullary rodding	Illizarov	Ilizarov + Intramedullary rod	Ilizarov fixation + intramedullary rod	Illizarov + Telescoping vascularized fibula
Site	Proximal tibia	Proximal tibia	Proximal tibia	Proximal tibia	Proximal tibia (transferred fibula)
Latency period (days)	7	10	5 to 8	7	8
Distraction rate	1 mm/day	1 mm/day (0.25 mm*4 times) then reduced to 0.5 mm/day	1 mm/day (0.5 mm*4 times)	NM	1 mm/day
Length achieved (cm)	3.8 cm	5.44 cm	3.7 cm	3–7 cm	2.3 cm
External fixation time	NM	186.4 days (lengthening days 92.8)	NM	NM	NM
Healing index	64 days/cm	NM	89 days/cm	NM	NM
Postop shortening (cm)	1.9	NM	NM	1.7	2.7 cm
Total follow-up (years)	4.5yrs	136.4 months	NM	5.2 years	10 years
Risk factors and complications	Delayed union (1 at bone transport site), Pin tract infection (15), Ankle stiffness (3), Equinus (2), Knee stiffness (2)	Regenerate fractures 4 (3 with NF), Pin tract infection 6 (5 had NF)	Proximal tibial dysplasia and repeat lengthening surgery are risk factors for distraction osteogenesis in CPT	Pin tract infection (number not mentioned)	Stress fractures 9, tibial shaft deformities 8
Remarks	Ilizarov used for compression if shortening was < 2.5 cm and for bone transport if shortening was > 2.5 cm	12/18 with NF had good callus, 6/18 poor callus	Divided into two groups based on healing index. Group 1: healing index < 65days/cm, Group 2: healing index > 65days/cm	Nil	Nil

*N *Number of patients, *NM* Not Mentioned, *NSTL* Not Specific for Tibial Lengthening, *BT *Bone Transport, *L *Lengthening

**Table 2:** Summary of extracted data

Author	El-Mowafi, H. et al [11]	El-Rosasy MA [12]	Enemudo RE et al [13]	Ghanem I et al [14]	Grill F [15]	Horn, J. et al [16]
Year	2005	2020	2023	1997	1996	2013
Region	Egypt	Egypt	Nigeria	France	Austria	Norway
Design	Retrospective	Retrospective	Retrospective	Retrospective	Retrospective	Retrospective
Duration	1997 to 2004	2011–2016	2017–2022	1985 and 1993	1983–1993	1987–2006
N	6 (BT 2; L 4)	25 (BT/L not mentioned)	5 (BT None; L 5)	14 (BT 14; L None)	9 (BT None; L 9)	15 (BT 6; L 9)
Age of lengthening	6.8 years	15 months to 15 years	11.6years	8.9 years	12.2 years	NM
Subgroups	NA	NA	NA	NA	NA	NA
NF-1	NM	24	5	11	5	14
Preop shortening (Mean)	5 cm	2.8 cm	7.2 cm	3.2 cm	NM	NM
Fixation method	Illizarov	Illizarov	Illizarov	Illizarov	Illizarov	Illizarov
Site	Proximal tibia	Proximal tibia	Proximal tibia	Proximal tibia	Proximal tibia	Proximal tibia
Latency period (days)	5	NM	7	NM	NM	NM
Distraction rate	1 mm/day (0.25 mm* 4 times)	NM	2 mm/day (360°−12 hourly)	NM	NM	NM
Length achieved (cm)	5 cm	6 cm	7 cm	NM	6.33 cm	4.2 cm
External fixation time	6.4 months	19 weeks	NM	7.8 months	7.9months	NM
Healing index	NM	NM	NM	NM	37.8 days/cm	NM
Postop shortening (cm)	0.5 cm	2.5 cm	0	NM	NM	NM
Total follow-up (years)	3.2 years	36.9 months	NM	3.5 years	4 years	6.7 years
Risk factors and complications	Premature consolidation 1, Pin tract infection (Number not mentioned)	Fracture of regenerate, Pin tract infection (Number not mentioned)	NM	NSTL	Fracture through ring fixator pin site in one case	Fracture of regenerate (7)
Remarks	Nil	Nil	Nil	Nil	Nil	Nil

*N *Number of patients, *NM* Not Mentioned, *NSTL* Not Specific for Tibial Lengthening, *BT *Bone Transport, *L* Lengthening

**Table 3:** Summary of extracted data

Author	Kristiansen LP et al [17]	Li Y et al [18]	Mathieu L et al [5]	Ohnishi I et al [19]	Vanderstappen, J. et al [20]	Yan A et al [21]
Year	2003	2024	2008	2005	2015	2017
Region	Norway	China	France	Japan	Belgium	China
Design	Retrospective	Retrospective	Retrospective	retrospective	Retrospective	Retrospective
Duration	1993–1995	2013 and 2022	1996 and 2005	1980 and 2000	1974 and 2007	2007 and 2010
N	7 (BT None; L 7)	14 (BT None; L 14)	5 (BT 5; L None)	32 (BT: 21; L 11)	12 (BT 12; L None)	8 (BT 8; L None)
Age of lengthening	3.2 years	33.7 years	Not mentioned for subgroup	Not mentioned for subgroup	Not mentioned for subgroup	NA
Subgroups	NA	NA	NA	NA	NA	NA
NF-1	7	7	NA	NA	5	Not mentioned for subgroup
Preop shortening (Mean)	NM	11.3 cm	NM	2.5 cm	NM	5 cm
Fixation method	Illizarov	Illizarov	Illizarov + intramedullary rod	Illizarov + intramedullary rod	Illizarov	Ilizarov + intramedullary rod
Site	Proximal tibia	Proximal tibia	Proximal tibia	Proximal tibia	Proximal tibia	Proximal tibia
Latency period (days)	7	5 to 7	not mentioned	NM	NM	NM
Distraction rate	1 mm/day (0.25 mm*4times)	0.75 to 1 mm/day	1 mm/day	NM	NM	NM
Length achieved (cm)	5.4 nm	10.2 cm	NM	Lengthening: 5.7 cm Transport: 4.3 cm	NM	NM
External fixation time	8.7 months	19.5 months	8.3 months	NM	NM	Not mentioned specifically for subgroup
Healing index	1.7 months/cm	NM	NM	NM	NM	NM
Postop shortening (cm)	0.46 cm	1.1 cm	NM	3.2 cm	0 cm	NM
Total follow-up (years)	not mentioned	26.8 months	4 years	6.7 yr	24 years	NM
Risk factors and complications	Refracture, knee contracture - number not mentioned	NSTL	NSTL	Pin tract infection, Poor regenerate (1)	NSTL	Pintract infection (number not mentioned for subgroup)
Remarks	Complications more frequent In younger populations	Nil	Nil	Multicenter study from 32 hospitals	Nil	Nil

*N* Number of patients, *NM* Not Mentioned, *NSTL* Not Specific for Tibial Lengthening, *BT* Bone Transport, *L* Lengthening

**Table 4:** Summary of extracted data

Author	Zayda AI et al [22]	Zhu GH et al [23]	Zhu GH et al [24]	Jang WY et al [25]	Kocaoğlu M et al [26]	Liu Y et al [27]
Year	2022	2015	2016	2019	2020	2023
Region	Egypt	China	China	Korea	Turkey	China
Design	Retrospective	Retrospective	Retrospective	Retrospective	Retrospective	Retrospective
Duration	2005 and 2018	2007 and 2012	2007 and 2010	1989–2013	1997 and 2017	2018–2020
N	16 (BT 14; L 2)	11 (BT 11; L None)	56 (BT 8; L Not mentioned)	12 (BT None; L 12)	8 (BT None; L 8)	9 (BT None; L 9)
Age of lengthening	5.4 years	8.5 years	Not mentioned for subgroup	Group 1–10.8years, Group 2–11.5years	5.9 years	9.4 years
Subgroups	NA	NA	NA	Group 1-Metadiaphyseal lengthening; Group 2-Physeal or subphyseal lengthening	NA	NA
NF-1	6	10	Not mentioned for subgroup	Not mentioned for subgroup	5	6
Preop shortening (Mean)	3.6 cm	5.6 cm	3.8 cm	NM	2.5 cm	NM
Fixation method	Illizarov + intramedullary rod	Illizarov	illizarov + intramedullary rod	Illizarov	Illizarov + intramedullary rod	Illizarov
Site	Proximal tibia	Proximal tibia	Proximal tibia	Meta-diaphyseal lengthening (5) (Group 1), Physeal or subphyseal lengthening (7) (Group 2)	Physis (3), Proximal tibial (5)	Physis
Latency period (days)	5 to 7	7	7	Physeal distraction 6, Sub- Physeal Distraction 4–5, Proximal tibia-not mentioned	NM	4
Distraction rate	1 mm/day (0.25 mm* 4 times)	0.5 mm/day; (0.25 mm/day for poor regenerate)	0.5 mm/day	Physeal distraction-1.5 mm/day for 4 days; physis reduced; After 2 days- 0.5 mm/day, Subphyseal-0.5 mm to 1 mm/day	NM	1 mm/day (0.25 mm*4times)
Length achieved (cm)	NM	5.3 cm	NM	Group 1: 3 cm, 4.2 cm Group 2: 4.2 cm	5 cm	6.6 cm
External fixation time	8.6 months	not mentioned	NM	NM	NM	316 days
Healing index	NM	63.1 days/cm (proximal tibial dysplasia), 52.7 days/cm (without dysplasia)	NM	83.3 days/cm in group 1, 35days/cm in group 2	NM	51.1 d/cm
Postop shortening (cm)	1.5 cm	NM	Not mentioned for subgroup	NM	1 cm	NM
Total follow-up (years)	NM	41 months	1.6 years	15.5years in group 1 and 10.5 years in group 2	8.5 yr	36.1 months
Risk factors and complications	Procurvatum deformity (4), Recurvatum (1), Pin tract infection all cases	Ankle joint stiffness (2), Pin tract infection (5)	Pin tract infection (6)	Pin tract infection in 4 patients, premature physeal arrest in 1 PDO, 3 patients had poor regenerate	NSTL	Pin tract infection 1, Ankle stiffness 1, Angular tibial deformity 1, Knee ROM limitation 1
Remarks	Nil	8 cases had proximal tibial dysplasia, poor regenerate in all cases with proximal tibial dysplasia	Nil	Physeal and Sub Physeal Distraction Osteogenesis superior to proximal tibial distraction osteogenesis in proximal tibial dysplasia	Nil	Nil

*N* Number of patients, *NM* Not Mentioned, *NSTL* Not Specific for Tibial Lengthening, *BT *Bone Transport, *L* Lengthening

**Table 5:** Summary of extracted data

Author	Paley D et al [2]	Vlad C et al [28]	Boero S et al [29]	Borzunov DY et al [30]
Year	1992	2013	1997	2022
Region	USA	Romania	Italy	Russia
Design	Retrospective	Retrospective	Retrospective	Retrospective
Duration	NM	2001 and 2011	NM	NM
N	12 (BT None; L 12)	7 (8 lengthenings)	16 (BT/L not mentioned)	9(BT 9/L None)
Age of lengthening	Not mentioned for subgroup	5.4 years	8.8years	6.1 years
Subgroups	NA	NA	NA	NA
NF-1	Not mentioned for subgroup	6	21	5
Preop shortening (Mean)	NM	NM	6.3 cm	6.0 cm
Fixation method	Ilizarov	Ilizarov	Illizarov	Ilizarov and induced membrane technique
Site	Proximal tibia (8), Physis (2), Pseudoarthrosis (2)	Physis	Proximal tibia, pseudoarthrosis site lengthening	Proximal tibia (8), proximal and distal tibia (1)
Latency period (days)	NM	NM	NM	5 to 7
Distraction rate	Physis − 0.5 mm/day, Proximal tibia-1 mm/day, Pseudoarthrosis 0.5 mm/day; then 1 mm/day after bone formation is noted	1 mm/day (0.25 mm*4times)	NM	1 mm/day, reduced to 0.5–0.75 mm based on regenerate
Length achieved (cm)	1.5 to 8 cm	6.6 cm	4.94 cm	2.6 cm
External fixation time	NM	NM	NM	70.1 days (lengthening days 31.9)
Healing index	NM	24 days/cm	NM	NM
Postop shortening (cm)	NM	NM	1.9 cm	NM
Total follow-up (years)	4 years	5 yr	2years	25.3 months
Risk factors and complications	1 delayed union of corticotomy, rest not specific to lengthening	Refracture at pseudoarthrosis site, no complications at lengthening site	Pin tract infection 4, supracondylar femur fracture 3, tibial valgus 4, tibial anterior bowing 1	Pin tract infection 4, Regenrate deformity 1, Poor regenerate 2
Remarks	Nil	Nil	Nil	Nil

*N* Number of patients, *NM* Not Mentioned, *NSTL* Not Specific for Tibial Lengthening, *BT* Bone Transport, *L* Lengthening

**Table 6:** Summary of extracted data

Author	Choi IH et al [31]	Gilbert A et al [7]	Broeking, J.*N*. et al [32]	Pirwani et al [33]
Year	2011	1995	2017	2013
Region	Korea	France	Germany	Pakistan
Design	Retrospective	Retrospective	Retrospective	Retrospective
Duration	1989–2007	1977–1993	NM	2008–2012
N	13 (BT/L not mentioned)	29 (BT None; L 29)	39 (BT 11; L 28)	9 (BT None; L 9)
Age of lengthening	Group-1 6.3years, Group 2–3.2years	5.5 years	9.4years	10–12 years
Subgroups	Group 1–4 in 1 osteosynthesis; Group 2-Other modalities	NA	Group A-Bone transport; Group B-Extrafocal lengthening	NA
NF-1	11	22	NM	5
Preop shortening (Mean)	NM	3.5 cm	NM	NM
Fixation method	Illizarov + intramedullary rod	Monolateral-Wagner technique of lengthening	Ilizarov + Intramedullary rod	Illizarov
Site	Bifocal compression distraction or bone transport	Proximal tibia		Physis
Latency period (days)	NM	NM	Proximal tibia	5
Distraction rate	NM	NM	NM	0.5 mm/day for 10 days; then 1 mm/day (0.25 mm*4times)
Length achieved (cm)	Group 1 (7 patients): 4.2 cm Group 2 (3 patients): 3 cm	NM	NM	10–12 cm
External fixation time	NM	NM	Group A: 7.3 cm Group B: 5.4 cm	13 months
Healing index	NM	NM		NM
Postop shortening (cm)	NM	1.7 cm	NM	NM
Total follow-up (years)	7–13yr	11.5 years	NM	12-48months
Risk factors and complications	Fracture of regenerate (Number not mentioned)	NSTL	NM	Pin tract infection 9
Remarks	Nil	Two patients had recurrence of shortening of 6 cm and 11 cm	NM	Nil

*N* Number of patients, *NM* Not Mentioned, *NSTL* Not Specific for Tibial Lengthening, *BT *Bone Transport, *L* Lengthening

### Characteristics of the included studies

All 31 studies in this review were retrospective in design, with publication years ranging from 1991 to 2023. The time frame at which these studies were performed ranged from 1989 to 2022. The articles were spread across Asia [5], Europe [15], Africa [11] and North America [1], with sample sizes ranging from 5 to 56 patients.

### Patient characteristics

A total of 486 patients were analyzed in this review.

The mean age at which tibial lengthening surgery was performed was mentioned in 24 studies. Among these, in 23 studies, lengthening was performed in skeletally immature children, and one study investigated the results of lengthening in skeletally mature patients. The mean age of lengthening at different sites in the proximal tibia has been presented in Table 7.

**Table 7:** Mean age of lengthening at sites above the proximal tibia

Site of lengthening	Age (Mean in years)
Skeletally immature, proximal tibial lengthening	7.8 (range: 3.2–10.8)
Skeletally immature, physeal or subphyseal lengthening	8.64 (range: 5.9–11.5)
Skeletally mature, proximal tibia	33.7

Twenty-one studies (number of CPT children: 278) investigated the association with neurofibromatosis type 1 (NF-1). Among these patients, 195 patients had NF-1 (70.14%).

The mean baseline tibial shortening was studied in 17 studies and was observed to be 4.9 cm (range: 2.5–11.3 cm).

### Surgical techniques

The Ilizarov ring fixator was the most common modality used for tibial lengthening. It was used as an isolated modality in 17 studies, with augmentation by an intramedullary rod in 11 studies, with telescoping of a vascularized fibula graft in 1 study and with the induced membrane technique in 1 study. A uniplanar fixator was used for tibial lengthening in 1 article.

The proximal tibia was the most common site used for lengthening in 22 studies. The other sites of tibial lengthening are listed in Table 8.

**Table 8:** Describing sites of lengthening over the tibia

Site of lengthening	Number of studies
Proximal tibia	22
Physis	3
Proximal tibia/Physis/Subphyseal region	1
Proximal tibia/Physis/Pseudoarthrosis site	1
Proximal tibia/Pseudoarthrosis	1
Proximal tibia/Physis	1
Proximal tibia/Bifocal proximal and distal tibia	1
Bifocal osteotomy	1

A total of 417 patients (85%) underwent lengthening through a proximal tibial osteotomy. Thirty-four (7%) children underwent lengthening through the physis. Fourteen children underwent lengthening through bifocal proximal and distal tibial osteotomies. A small subset of children underwent lengthening through the pseudoarthrosis site. In one study, lengthening was performed either at the proximal tibia or the pseudoarthrosis site, but the sample size of each subgroup was not mentioned.

The latency period between osteotomy and distraction ranged from 5 to 10 days.

The most common rate of distraction was 1 mm/day for proximal tibial osteotomy and 0.5 mm/day for lengthening through the physis.

### Outcomes of tibial lengthening

The reported means (length achieved, healing index, residual shortening) were derived from subsets of studies with heterogeneous definitions and incomplete reporting. These observations were not pooled or comparative results.

The mean overall length achieved was 5.5 cm (range 1.5–10.2 cm). When lengthening was performed at the physis, the mean length achieved was greater (7.1 cm) than when lengthening was performed at the proximal tibial level (5.34 cm).

Final tibial shortening after the lengthening procedure was mentioned in 14 studies. It was observed to be 1.4 cm (range 0–3.2 cm).

Nine studies described the healing index (time taken for consolidation per centimeter of regenerate/days) for tibial lengthening in CPT. The mean overall healing index was 50.2 days/cm (range 24–83.3 days/cm). Compared with proximal tibial distraction osteogenesis, physeal distraction (36.7 days/cm) was faster.

The overall follow-up period ranged from 1.6 to 24 years.

### Complications incurred and risk factors for tibial lengthening in CPT

In the included studies of the review, only the complications observed specifically during lengthening procedure were analyzed. Twenty-three studies described complications observed during the lengthening procedure. The most common complication observed was pin tract infection (14 studies, 60%) due to the external fixator pins. Other commonly mentioned complications include regeneration fractures, delayed consolidation of regenerate requiring additional procedures such as bone grafting, tibial shaft deformities at the lengthening site, stress fractures and adjacent joint stiffness (knee and ankle). In one child who underwent proximal tibial lengthening, early consolidation of the osteotomy site occurred, which required repeat osteotomy. In one child who underwent physeal distraction for tibial lengthening, permanent physeal arrest was observed.

Few studies have described risk factors such as proximal tibial dysplasia (defined as trumpet-shaped narrowing of the proximal tibia, anterior inclination of the proximal tibial physis, and anterior cortical concavity of the proximal tibia [3]) as major risk factors for proximal tibial lengthening. Some articles mentioned repeat lengthening procedures and early age at surgery as potential risk factors.

These complications and risk factors were reported heterogenously as observations during the lengthening procedure, not established or generalizable risk factors.

## Discussion

In this scoping review, 31 studies with 486 patients over a 33-year time frame covering a large geographical distribution were investigated to map the evidence available on tibial lengthening in the CPT. No similar review has been performed in the literature reviewed.

Many studies described strategies to achieve union at nonunion sites. However, tibial shortening was a persistent problem with long-term consequences if left unaddressed. Tibial lengthening as a widely used method to address this issue.

### Surgical techniques

The Ilizarov fixator in isolation [2, 6, 11–18, 20, 25, 27, 28, 33] or with hybrid techniques such as intramedullary rods [1, 3, 5, 8, 19, 21, 22, 24, 26, 31] was the most common and widely used strategy to achieve target tibial lengths. This method, being versatile, was used to achieve union at the nonunion site as well as perform lengthening through a tibial osteotomy in conjunction with the primary procedure or after union was achieved. In the literature reviewed, both segmental bone transport and corticotomy and lengthening were strategies used to achieve length; the former used when large bone fragments (> 2.5 cm) were excised, and the latter used when less bone excision was needed (< 2.5 cm) [1].

### Site of lengthening in the tibia–proximal tibia and physis

Lengthening was performed at various sites on the tibia. Proximal tibial osteotomy and lengthening [1, 3–6, 8, 11–24, 33] were the most common sites, followed by physeal or subphyseal distraction [2, 25–29]. The decision on the location of the osteotomy was based on proximal tibial dysplasia evident on radiographs [3]. Osteotomy performed through the dysplastic proximal tibia had delayed healing rates owing to the presence of pathological tissue at the osteotomy site [3]. Hence, physeal distraction was used as an alternate modality in this setting [25]. Physeal distraction required more technical expertise than proximal tibial osteotomy [25, 33]. The usual protocol followed was an acute distraction phase lasting for 4‒5 days until physis separation was visible on radiographs and the patient experienced acute pain at the proximal tibia. The physis was then gradually reduced for 2–3 days, followed by gradual lengthening.

Distraction at the proximal tibia was commonly performed at a rate of 1 mm/day versus slower distraction rates of 0.5 mm/day for physeal lengthening. This rate of distraction was apt for osteogenesis at the distraction site [6]. 

Overall, a length of 5.5 cm was achieved with method of distraction osteogenesis in CPT. The length achieved by physeal distraction was greater than that achieved by proximal tibial lengthening, with faster healing rates owing to the growth potential and vascularity at the physis. Physeal lengthening carried the risk of permanent physeal arrest; hence, it was recommended that it be performed closer to skeletal maturity to avoid this complication [25].

### Complications

Lengthening during CPT was fraught with complications. There were high rates of pin tract infections due to the longer duration of external fixator use. However, it could be managed with regular cleaning and dressing of the pin sites. Few patients required debridement or pin exchange. There was a theoretical risk of septic arthritis when distraction was performed at the physis due to the intra-articular nature of the pins. However, this phenomenon was not reported in any study.

Other complications included regenerate fractures and tibial bowing deformities, reflecting the biological challenges in the treatment of CPT.

### Strengths of tibial lengthening in CPT

Tibial lengthening during CPT provided effective correction of the limb length discrepancy. It could be combined with union-promoting techniques such as intramedullary rodding or bone grafting with reasonable success. This approach provided added advantages of simultaneous deformity correction during the distraction process.

### Limitations and challenges of tibial lengthening in CPT

Several clinically relevant factors should be considered while considering lengthening in congenital pseudoarthrosis of tibia. Pin tract infections were the most commonly reported complication which required treatment with antibiotics, debridement or pin exchange depending on the severity of infection.

Refracture at the pseudoarthrosis or at the regenerate site remained a common complication especially after frame removal or during subsequent growth indicating biological bone fragility and prolonged mechanical stress on the bone.

Residual angular or rotational deformities of the tibia occasionally could persist despite initial successful union and lengthening which could impact long term joint or limb biomechanics.

Furthermore, repeated surgeries and lengthening procedures could pose significant psychosocial burden due to disruptions in schooling or family life, but is inconsistently reported in the reviewed literature.

Consideration of these factors is essential for balanced interpretation of outcomes and for appropriate preoperative counseling of the child and family.

### Research gaps

This scoping review was dominated by small, single-center, retrospective studies without standardized functional outcome measures. Prospective, multicenter studies would aid in generalizing the results across heterogeneous population groups. Studies assessing patient-reported outcomes and quality of life would add value in planning treatment for patients with CPT. The evaluation of newer limb lengthening technologies with a large cohort of patients is the need of the hour in CPT.

### Limitations of the review

The exclusion of case series with fewer than five patients, which may have introduced selection bias, particularly in the analysis of complications in this rare condition. This criterion was applied to improve data consistency across studies in an otherwise heterogenous literature rather than to discount smaller reports.

All included publications were retrospective in nature with heterogeneous outcome definitions. There were no studies that described functional or patient reported outcomes.

## Conclusions

In CPT, limb length equalization by tibial lengthening is an important adjunct in the armamentarium of the treating surgeon. However, the complication rates are high. Refracture, need for repeated lengthening, residual deformity and prolonged treatment duration remain significant challenges, particularly in biologically compromised bone. The available evidence is heterogenous and largely derived from single center retrospective studies, limiting definitive conclusions regarding optimum techniques and outcomes or risk-benefit balance. Careful patient selection and thorough preoperative counseling are paramount to achieve favourable outcomes and mitigate psychosocial burden on treatment. Standardization of reporting outcomes and collaborative prospective studies are needed to optimize patient outcomes and establish best practices.

## Supplementary Information

Supplementary Material 1.

Supplementary Material 2.

Supplementary Material 3.

## Data Availability

The datasets used and/or analyzed during the current study are available from the corresponding author upon reasonable request.

## Abbreviations

CPT: Congenital Pseudoarthrosis of Tibia
