# Supplementary material for: Tibial lengthening in congenital pseudoarthrosis of the tibia: a scoping review
Source: BMC Musculoskelet Disord. 2026 Mar 6;27:307. doi: 10.1186/s12891-026-09666-4 (PMC13078090; doi:10.1186/s12891-026-09666-4)
Supplement: Supplementary file 1 — Supplementary Material 1. [file 12891_2026_9666_MOESM1_ESM.docx]

**Search strategy (PubMed): June 2025**

| **No.** | **Concept** | **Query** | **Result** |
| --- | --- | --- | --- |
| **#1** | **Tibial lengthening**  (tibial lengthening OR limb lengthening OR tibial length equalization OR Ilizarov technique OR distraction osteogenesis) | **"tibial lengthening"[All Fields] OR "limb lengthening"[All Fields] OR (("tibia"[MeSH Terms] OR "tibia"[All Fields] OR "tibial"[All Fields] OR "tibialization"[All Fields] OR "tibially"[All Fields] OR "tibials"[All Fields]) AND ("length"[All Fields] OR "lengths"[All Fields]) AND ("equal"[All Fields] OR "equaled"[All Fields] OR "equaling"[All Fields] OR "equalisation"[All Fields] OR "equalise"[All Fields] OR "equalised"[All Fields] OR "equalises"[All Fields] OR "equalising"[All Fields] OR "equalities"[All Fields] OR "equality"[All Fields] OR "equalization"[All Fields] OR "equalizations"[All Fields] OR "equalize"[All Fields] OR "equalized"[All Fields] OR "equalizer"[All Fields] OR "equalizers"[All Fields] OR "equalizes"[All Fields] OR "equalizing"[All Fields] OR "equalled"[All Fields] OR "equalling"[All Fields] OR "equally"[All Fields] OR "equals"[All Fields])) OR "Ilizarov technique"[All Fields] OR "distraction osteogenesis"[All Fields]** | **9764** |
| **#2** | **Congenital pseudoarthrosis tibia**  (congenital pseudoarthrosis tibia OR congenital pseudoarthrosis OR congenital pseu*arthrosis) | **"congenital pseudoarthrosis tibia"[All Fields] OR "congenital pseudoarthrosis"[All Fields] OR "congenital pseu*arthrosis"[All Fields]** | **745** |
| **#1 AND #2** |  | **("congenital pseudoarthrosis tibia"[All Fields] OR "congenital pseudoarthrosis"[All Fields] OR "congenital pseu*arthrosis"[All Fields]) AND ("tibial lengthening"[All Fields] OR "limb lengthening"[All Fields] OR (("tibia"[MeSH Terms] OR "tibia"[All Fields] OR "tibial"[All Fields] OR "tibialization"[All Fields] OR "tibially"[All Fields] OR "tibials"[All Fields]) AND ("length"[All Fields] OR "lengths"[All Fields]) AND ("equal"[All Fields] OR "equaled"[All Fields] OR "equaling"[All Fields] OR "equalisation"[All Fields] OR "equalise"[All Fields] OR "equalised"[All Fields] OR "equalises"[All Fields] OR "equalising"[All Fields] OR "equalities"[All Fields] OR "equality"[All Fields] OR "equalization"[All Fields] OR "equalizations"[All Fields] OR "equalize"[All Fields] OR "equalized"[All Fields] OR "equalizer"[All Fields] OR "equalizers"[All Fields] OR "equalizes"[All Fields] OR "equalizing"[All Fields] OR "equalled"[All Fields] OR "equalling"[All Fields] OR "equally"[All Fields] OR "equals"[All Fields])) OR "Ilizarov technique"[All Fields] OR "distraction osteogenesis"[All Fields])** | **147** |
