# Supplementary material for: Tibial lengthening in congenital pseudoarthrosis of the tibia: a scoping review
Source: BMC Musculoskelet Disord. 2026 Mar 6;27:307. doi: 10.1186/s12891-026-09666-4 (PMC13078090; doi:10.1186/s12891-026-09666-4)
Supplement: Supplementary file 3 — Supplementary Material 3. [file 12891_2026_9666_MOESM3_ESM.docx]

| Author |  |
| --- | --- |
| Year |  |
| Region |  |
| Design |  |
| Duration |  |
| N |  |
| Age of lengthening |  |
| Sub-groups |  |
| NF-1 |  |
| Pre-op shortening (Mean) |  |
| Fixation method |  |
| Site |  |
| Latency period (days) |  |
| Distraction rate |  |
| Length achieved (cm) |  |
| External fixation time |  |
| Healing index |  |
| Post-op shortening (cm) |  |
| Total follow-up (years) |  |
| Risk factors and complications |  |
| Remarks |  |
